# Association between MRI findings and inflammatory symptoms in non-specific chronic low back pain

**DOI:** 10.1007/s00586-025-09492-7

**Published:** 2025-10-22

**Authors:** Katharina Ziegeler, Lianne S. Gensler, Thomas M. Link, Colin Roach, Aaron Wolfe Scheffler, Aaron J. Fields, Noah Bonnheim, Abel Torres-Espin, Jeffrey C. Lotz, Trisha Hue, Mark Wallace, Scott Fishman, Patricia Zheng, Conor O’Neill

**Affiliations:** 1 Department of Radiology and Biomedical Imaging, University of California, San Francisco, San Francisco, United States; 2 Department of Medicine, Division of Rheumatology, University of California, San Francisco, San Francisco, United States; 3 Department of Orthopaedic Surgery, University of California, San Francisco, San Francisco, United States; 4 Department of Epidemiology and Biostatistics, University of California, San Francisco, San Francisco, United States; 5 School of Public Health Sciences, University of Waterloo, Waterloo, Canada; 6 Department of Anaesthesiology, University of California, San Diego, San Diego, United States; 7 Department of Anaesthesiology and Pain Medicine, University of California, Davis, Davis, United States

**Keywords:** Low back pain, Inflammatory back pain, MR imaging, Lumbar spine

## Abstract

**Purpose:**

Non-specific chronic low back pain (nsCLBP) likely constitutes a heterogeneous group of conditions, and identifying an inflammatory phenotype may improve treatment stratification. The objective of this study was to determine associations between inflammatory back pain (IBP) features and MRI findings in subjects with nsCLBP.

**Methods:**

Participants were selected from the longitudinal Clinical Cohort for Comprehensive Deep Phenotyping of Chronic Low-Back Pain Adults Study (comeBACK), a cohort of adults with nsCLBP. IBP features (morning stiffness, nocturnal LBP, symptom improvement with exercise / worsening with rest, insidious onset, onset < 40 years) were assessed via questionnaire. MRI scans of the lumbar spine were interpreted by a radiologist, using a comprehensive scoring system: Modic changes (MC), endplate erosion, facet joint arthritis, central canal stenosis and degeneration of the sacroiliac joints (SIJ). Logistic regression was performed (presence IBP feature as dependent variable and MRI findings as independent variables).

**Results:**

A total of 290 individuals (159 female) were included. Both endplate erosion and MC1 changes were positively associated with overall IBP (erosion: OR 2.1, 95%CI 1.3–3.3; MC1: OR 2.2, 95%CI 1.4–3.4), and specifically morning stiffness and worsening with rest. Negative association with overall IBP symptoms was found for SIJ degeneration (OR 0.6, 95%CI 0.4-1.0) and no association for facet arthropathy, MC2 and central canal stenosis.

**Conclusion:**

Our exploratory analysis supports the notion of an inflammatory nsCLBP phenotype with distinguishing imaging features, by establishing associations between endplate erosion and Modic type 1 changes with select IBP features.

**Supplementary Information:**

The online version contains supplementary material available at 10.1007/s00586-025-09492-7.

## Introduction

Only a small minority of chronic low back pain (CLBP) patients have clearly defined pathology such as tumor, infection, fracture or inflammatory disease. CLBP in the absence of these disorders is classified as non-specific (nsCLBP) [1]. Evidence from randomized controlled trials (RCT) supports a number of interventions for nsCLBP, but the effect sizes are generally small [2–4]. As a result, many patients turn to unproven treatments that carry significant risks of harm, such as opioids and surgery. Developing better treatment strategies for nsCLBP is critical.

nsCLBP patients are heterogenous, with variable clinical characteristics, prognoses, and responses to treatment [5]. Identifying phenotypes that influence treatment response within this heterogenous disorder is a major focus of spine research [6]. Between 28 and 38% of individuals with CLBP have inflammatory back pain (IBP) [7], a clinical syndrome that includes varying degrees of morning stiffness, improved pain with exercise, worsened pain with rest, and night-time pain. IBP is a hallmark feature of axial spondyloarthritis (axSpA), a disorder of the immune system predominantly affecting the axial skeleton, typified by radiographic axSpA, also known as Ankylosing Spondylitis [8]. The estimated population prevalence of axSpA is 0.3% to 1.4%, far below the prevalence of IBP (5–6%) [7, 9]. Given these findings, a nsCLBP inflammatory, but non-immune-mediated, phenotype has been proposed, but there has been little research on the defining characteristics [7].

While magnetic resonance imaging (MRI) is widely used for evaluation of nsCLBP, the high prevalence of degenerative changes in asymptomatic subjects makes it difficult to discriminate between painful pathology and incidental age-related findings [10]. Defining the clinical relevance of MRI findings in individual nsCLBP patients is a critical unmet need. There are several imaging lesions which are plausible sources of inflammatory symptoms in nsCLBP. Of these, Modic type 1 lesions have the best evidence for an inflammatory pathogenesis [11, 12]. Associated with Modic lesions, and perhaps a more advanced stage of the same pathologic process, are erosions of the vertebral endplates [13]. Other potentially inflammatory lesions commonly seen on MRI are osteoarthritis (OA) of the facet joints and the sacroiliac (SI) joints. Both joints may exhibit signs of active synovitis, which if seen with OA of peripheral joints can be accompanied by clinical symptoms of inflammation [14]. Conversely, impingement of nerve structures, such as can be seen in (degenerative) stenosis of the central spinal canal and the neuroforamen, is generally more closely linked to neuropathic symptoms [15]. The combination of inflammatory symptoms and an inflammatory MRI lesion may constitute a clinically relevant nsCLBP phenotype, which can be used to select patients for treatments that specifically target spinal inflammation.

There is limited data on the association between IBP and specific imaging lesions. Bailly et al. found an association between Modic lesions and inflammatory pain pattern (defined as pain maximum in the morning, waking at night from pain and morning stiffness >60 min) and response to oral steroids [16]. Less is known about the association of other imaging findings with IBP features, and whether the association with Modic 1 is similar across different clinical features of IBP.

The aim of this study was to investigate the association between different MR imaging findings of the spine and individual IBP symptoms in a cohort of individuals with nsCLBP. Our hypothesis was that positive associations exist between IBP symptoms and Modic 1 lesions, endplate erosions and facet arthritis, with no association between IBP symptoms and central canal stenosis.

## Methods

### Study participants

The Longitudinal Clinical Cohort for Comprehensive Deep Phenotyping of Chronic Low-Back Pain Adults Study (comeBACK) is a longitudinal cohort of adults with nsCLBP, conducted by the University of California, San Francisco as part the NIH-funded Back Pain Consortium Research Program (BACPAC). Details of this cohort have been published elsewhere [17]. To be eligible, study participants must have CLBP, defined as pain between the lower posterior margin of the rib cage and the horizontal gluteal fold, which has persisted for at least the past 3 months and has resulted in pain on at least 50% of days in the past 6 months [18]. Subjects with cancer, infections, fractures, rheumatologic conditions (including SpA), serious neurologic disorders (e.g. foot drop), or are pregnant are excluded, to ensure a nsCLBP sample population. A total of 450 individuals were prospectively recruited in the comeBACK study – at the time of this analysis, 290 of these had received semi-quantitative assessments of spinal MR imaging (see section below) and had complete questionnaire data for inflammatory back pain symptoms and were therefore eligible for inclusion in this ancillary analysis. All patients gave informed consent to use of data for scientific purposes and institutional review board approval was attained before commencement of the study.

## Assessment of inflammatory back pain

Inflammatory back pain symptoms were measured using items from an arthritis questionnaire (ARQ) that was specifically developed and validated for the 2009–10 US National Health and Nutrition Examination Survey (NHANES) [7]. The purpose of the ARQ was to provide population-based prevalence estimates for four published IBP classification criteria: Calin [19], European Spondylarthropathy Study Group (ESSG) [20] and Berlin criteria sets 8a and 7b [21]. The ASAS IBP classification criteria were published after the NHANES study was fielded and were not included [22]. Items from the ARQ included in this study assessed the features ‘insidious onset’ (time till pain was fully developed; longer than 1 month as cut-off), ‘young age at onset of LBP’ (age < 40 years at symptom onset), ‘duration of morning stiffness’, ‘improvement of pain with exercise’ (e.g. walking or stretching for 30 min), ‘worsening of pain with rest’ (what is the effect of sleep/rest on back pain = worsening), and ‘night-time pain’ (waking from back pain after ≥ 4 h of sleep). To be consistent with the criteria adopted in the NHANES study morning stiffness had to last for 30 min or more to be classified inflammatory. Morning stiffness of more than 4 h was not classified as an IBP feature, because of possible overlap with nociplastic pain, or conditions such as fibromyalgia [23]. The original questionnaire is provided as an online appendix (**Appendix 1**).

## Spinal imaging

**Fig. 1:**
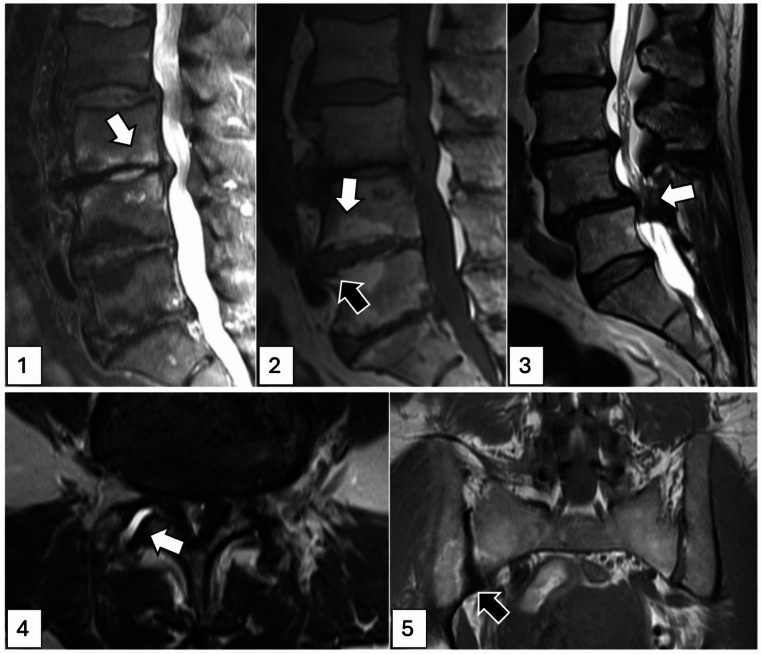
Imaging lesions. 1 = Mid-spinal section of sagittal T2 weighted MR image with fat saturation, showing Modic type 1 lesions (white arrow) in the L3/4 disc segment. 2 = Mid-spinal section of sagittal T1-weighted MR image, showing Modic type 2 lesions (white arrow) and endplate erosion (black arrow) in the L4/5 disc segment. 3 = Mid-spinal section of sagittal T2-weighted image without fat-saturation, showing severe central canal stenosis (white arrow) in the L4/5 disc segment. 4 = Axial T2-weighted image without fat-saturation, showing facet arthropathy with fluid in the right facet joint (white arrow). 5 = Coronal T1-weighted image showing degenerative SI joint changes on the right side with sclerosis (black arrow)

MRI scans of the lumbar spine were obtained on 3 Tesla scanners and included sagittal fat-saturated T2-weighted and T1-weighted spin-echo (SE) sequences as well as T1- and T2-weighted axial SE sequences and one coronal T1-weighted SE sequence. All scans were interpreted by a musculoskeletal radiologist (T.M.L, 27 years of experience), using an established scoring system [24], the REACH score. The REACH score is designed as a comprehensive assessment of the lumbar spine, including a wide array of pathologies (see **Appendix 2** for more detail). For this specific analysis, vertebral endplate bone marrow changes, vertebral endplate erosions and facet arthropathy were selected for further analysis, hypothesizing a positive association with IBP, as well as central canal stenosis as a control lesion, that was not expected to be associated with IBP. A visual guide to the assessment of these lesions is given in Fig. 1. Vertebral endplate bone marrow changes were classified according to Modic, where type 1 corresponds to increased fluid signal relative to normal bone marrow, i.e. bone marrow edema, and type 2 corresponds to increased fat signal [25]. Endplate erosions, defined as irregular, diffuse erosions of the vertebral endplates were graded as absent or present. Both Modic changes and endplate lesions were assessed at each vertebral endplate from the inferior endplate of L1 to the superior endplate of S1. Central canal stenosis was graded as absent, mild (mild constriction, minimal loss of fluid around rootlets), moderate (fluid diminished but present), or severe (complete loss of fluid) at each disc level from L1/2 to L5/S1; for the purpose of this analysis, only segments with at least moderate stenosis were counted as positive for stenosis. Facet arthropathy was also assessed at disc levels L1/2 to L5/S1, and presence of active facet arthropathy was assumed in the presence of both osteophytes and fluid within the joint space (on one or both sides). Degenerative changes of the sacroiliac joint (sclerosis or osteophytes) were assessed as present or absent.

### Statistical analysis

All analyses were performed using Stata Version 17. To investigate the association between IBP symptoms (outcome) and imaging features (predictors), logistic and ordinal regression analysis were performed, adjusted for age, sex and BMI, as a contributor to systemic inflammation. This analysis was performed at the patient level, meaning that patients were considered positive for an imaging finding if it was found in any of the locations described above. For ordinal logistic regression, modeling assumptions were assessed by comparing coefficients across cumulative binary logistic models, indicating acceptable stability for the main predictor, despite minor deviations for covariates (sex, BMI).

## Results

### Study subjects

A total of 290 participants had complete data and were included in the analysis, of which 2 subjects reported no IBP symptoms, 26 reported 1 IBP symptom, 68 reported 2 IBP symptoms, 87 reported 3 IBP symptoms, 63 reported 4 IBP symptoms, 31 reported 5 IBP symptoms, and 13 reported 6 IBP symptoms. The most reported IBP symptom was improvement of pain with exercise (81.0%; 235/290), followed by insidious onset (68.3%; 198/290), and age at onset under 40 years (59.7%; 173/290) with waking from pain reported by the fewest participants (30.0%; 87/290). Demographic characteristics are provided in Table 1.

**Table 1:** Demographics and imaging findings

			All (*n* = 290)
Age [mean years, SD]			53.3 (15.6)
Females [%,n]			54.8% (159/290)
Race [%,n]	AI/AN		2.1% (6/290)
	Asian		12.8% (37/290)
	Black/AA		7.2% (21/290)
	NH/PI		1.4% (4/290)
	White		76.6% (222/290)
	unknown		1.0% (3/290)
Mean BMI [kg/m^2^, SD]			26.4 (4.9)
Overweight to obese (BMI > 25) [%,n]			56.9% (165/290)
Central obesity* [%,n]			74.1% (215/290)
LBP duration [mean years, SD]			12.0 (12.4)
Current NSAID medication [%,n]			59.0% (171/290)
Current opioid medication [%,n]			7.9% (23/290)
Nicotine use (ever) [%,n]			35.9% (104/290)
Depression [mean t-score, SD]			48.2 (8.0)
Charlson co-morbidity index [median, IQR]			2 (3)
Pain at baseline			5 (3)
MRI findings [%,n]		Endplate erosion	52.8% (153/290)
		Modic type 1	46.6% (135/290)
		Modic type 2	36.9% (107/290)
		Facet arthropathy	37.6% (109/290)
		SIJ changes	35.2% (102/290)
		Central canal stenosis	29.3% (85/290)

SD = standard deviation. AI/AN = American Indian or Alaska Native. AA = African American. NH/PI = Native Hawaiian or Pacific Islander. LBP = low back pain. *=central obesity defined by waist-to-height ratio > 0.5. NSAID = non-steroidal anti-inflammatory drugs. Depression = mean t-score on PROMIS depression scale. IQR = inter-quartile range. MRI = magnetic resonance imaging. SIJ = Sacroiliac joint

## Frequency of imaging findings

**Fig. 2:**
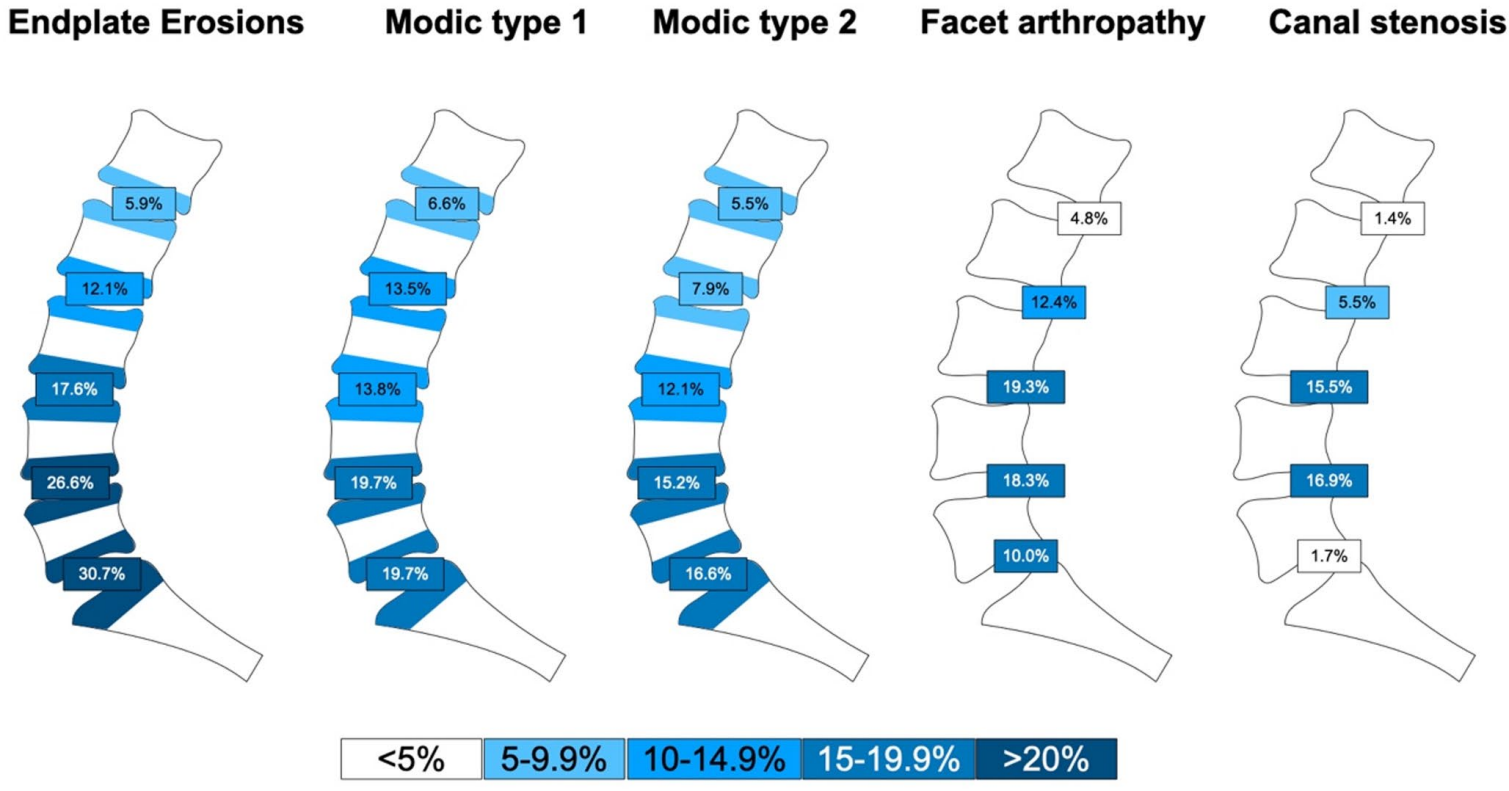
Distribution of MRI lesions. Relative frequencies of imaging lesions per lumbar spine level

The distributions of endplate erosion and endplate-associated bone marrow changes, facet arthropathy and central canal stenosis are shown in Fig. 2. Endplate erosions, Modic type 1 lesions, and Modic type 2 lesions all followed a cranio-caudal gradient with the lowest prevalence at L1/2 and highest prevalence at L5/S1. Canal stenosis was most common at L4/5, and rare at L5/S1. Degenerative SIJ changes were observed in 35.2% (102/290) subjects. Endplate erosions were observed in 52.8% (153/290) subjects, but only a minority of affected individuals exhibited such lesions in more than two spinal segments (11.7% 34/290). Similar observations were made for Modic type 1 lesions, which were seen in 46.6% (135/290) of subjects, of which only 6.2% (18/290) had such lesions in more than two spinal levels. Modic type 2 lesions were slightly less prevalent (36.8%;107/290) but multilevel disease (i.e. manifestation in more than two levels) was similarly rare (4.1%; 12/290). Active facet arthropathy was found in 37.6% (109/290) subjects with multilevel disease in 6.2% (18/290). Central canal stenosis (moderate or severe) was seen in 29.3% (85/290) of patients, with multilevel changes in only 1.7% (5/290).

## Association of imaging findings and inflammatory symptoms

To explore the associations between inflammatory symptoms and imaging markers, separate logistic regressions were performed for each IBP symptom as predictor, and imaging markers as outcomes. Furthermore, an ordinal logistic regression, with number of IBP symptoms as outcome, was performed. Results of these analyses are summarized in Table 2. Presence of endplate erosions was positively associated with fulfilment of more IBP symptoms (OR 2.1, *p* = 0.001), morning stiffness (OR 2.7, *p* < 0.001) and worsening of pain with rest (OR 2.2, *p* = 0.004). Presence of Modic type 1 lesions were positively associated with a greater number of inflammatory symptoms (OR 2.2, *p* < 0.001). This positive association was likely driven by positive associations with morning stiffness and worsening with rest, with ORs of 2.2 (*p* = 0.002) and 2.5 (*p* < 0.001), respectively. Facet arthritis was neither positively nor negatively associated with IBP symptoms or features. Degenerative changes of the SI joints were negatively associated with a higher number of IBP symptoms with OR 0.6 (*p* = 0.033) while neither positive nor negative associations were found for central canal stenosis. An additional table showing associations between IBP and clinical features is provided as **Appendix 3**.

**Table 2:** Association between imaging lesions and clinical IBP features

		Endplate erosion	Modic type 1	Modic type 2	Facet arthropathy	SIJ changes	Central canal stenosis
**IBP feature [OR**,** 95%CI**,** p]**	Sum of IBP symptoms	2.1* **(1.3; 3.3)** *p* = 0.001	2.2* **(1.4; 3.4)** *p* < 0.001	0.9 (0.6; 1.5) *p* = 0.747	1.1 (0.7; 1.8) *p* = 0.602	0.6* **(0.4; 1.0)** *p* = 0.033	0.8 (0.5; 1.3) *p* = 0.373
	Morning stiffness	2.7* **(1.6; 4.6)** *p* < 0.001	2.2* **(1.3; 3.6)** *p* = 0.002	0.8 (0.5; 1.4) *p* = 0.461	0.9 (0.5; 1.5) *p* = 0.579	1.3 (0.8; 2.3) *p* = 0.308	0.8 (0.4; 1.3) *p* = 0.333
	Improvement with exercise	1.7 (0.9; 3.1) *p* = 0.118	1.8 (0.9; 3.3) *p* = 0.076	1.4 (0.7; 2.7) *p* = 0.313	1.2 (0.6; 2.3) *p* = 0.565	0.8 (0.4; 1.5) *p* = 0.491	0.8 (0.4; 1.5) *p* = 0.440
	Worsening with rest	2.2* **(1.3; 3.8)** *p* = 0.004	2.5* **(1.5; 4.2)** *p* < 0.001	0.8 (0.5; 1.4) *p* = 0.495	1.1 (0.7; 1.9) *p* = 0.688	0.7 (0.4; 1.2) *p* = 0.162	0.7 (0.4; 1.3) *p* = 0.258
	Waking from pain	1.0 (0.6; 1.7) *p* = 0.986	1.4 (0.9; 2.4) *p* = 0.172	1.0 (0.6; 1.7) *p* = 0.916	0.9 (0.5; 1.6) *p* = 0.718	0.6 (0.4; 1.2) *p* = 0.138	1.1 (0.6; 2.0) *p* = 0.809
	Younger than 40 years old at onset	1.4 (0.7; 2.5) *p* = 0.324	1.0 (0.5; 1.7) *p* = 0.895	1.1 (0.6; 2.0) *p* = 0.778	1.1 (0.6; 2.1) *p* = 0.660	0.7 (0.3; 1.2) *p* = 0.182	1.3 (0.7; 2.5) *p* = 0.459
	Insidious onset	0.9 (0.5; 1.5) *p* = 0.614	1.1 (0.6; 1.8) *p* = 0.810	0.9 (0.5; 1.5) *p* = 0.582	1.5 (0.8; 2.6) *p* = 0.167	0.7 (0.4; 1.2) *p* = 0.164	0.7 (0.4; 1.4) *p* = 0.346

Results of ordinal (for sum of IBP symptoms) and logistic regression analyses, with IBP feature as outcome, and respective imaging lesion as predictor, adjusted for age, sex and BMI. Log-odds from ordinal regression are transformed to ORs for ease of interpretation. Statistically significant results (*p* < 0.05) are printed in bold and marked with as asterisk (*)

## Discussion

Our exploratory study investigated the associations between IBP features and different imaging findings in a prospectively recruited cohort of adults with nsCLBP. We found positive associations between specific IBP features (morning stiffness and symptom worsening with rest) and both endplate erosions and Modic type 1 lesions. These data support our hypothesis that there is a degenerative inflammatory phenotype, defined by a combination of symptoms and MRI findings. The association between erosive endplate changes and pain has not been detailed in the literature thus far, and may reflect disc-vertebra crosstalk, which has been demonstrated in surgical tissue and animal models [26].

Our results are consistent with those of Bailly, et al. who found, in a case control study, that patients with Modic 1 lesions more frequently exhibited an inflammatory pain pattern, as well as worse pain with lumbar extension, and responded better to oral steroids than did controls [16]. Our study differs from theirs mainly in the separate analysis of different IBP features, and a stricter definition of IBP overall. Our findings also support those of Arnbak et al., who found positive associations between vertebral endplate bone marrow edema and stiffness [27].

Our study is the first to establish a connection between endplate erosions and IBP symptoms. The association of erosive endplate changes with inflammatory symptoms supports the disc/bone marrow cross-talk theory [26]. According to this theory, based on research using both animals and human surgical tissue samples [28], endplate damage increases communication between the bone marrow and degenerated discs, which produce a number of inflammatory mediators, which leads to fibrogenic and pro-inflammatory cross-talk between MC bone marrow and adjacent discs [11].

Contrary to our original hypothesis, facet arthropathy showed only sparse associations with IBP symptoms; these findings were surprising in light of the evidence from osteoarthritis research, that links joint inflammation in degenerative joint disease to inflammatory symptoms [29]. Similarly unexpected were the significant negative associations between degenerative SIJ changes and IBP symptoms. While patients with sacroiliitis were deliberately excluded from this study, the findings are nevertheless surprising, because IBP symptoms, while less common than in patients with axSpA, can be found in up to 39.5% of patients with primarily degenerative conditions of the SI joints [30].

All the different IBP classification criteria sets (Calin, Berlin, ESSG, and ASAS) have been developed using expert opinion (considering other criteria such as imaging findings and HLA-B27) as the reference standard for axSpA. As there is no reference standard for spinal inflammation in nsCLBP, it is not possible to rigorously test the assumption that IBP symptoms in that condition correspond to inflammation in spinal tissues. Given these different criteria sets we used a pragmatic approach for defining the IBP criteria in this study, keeping patient burden in mind. While the value of morning stiffness in discriminating between axSpa and nsCLP has been questioned [27], we elected to retain this, using the 30 min duration threshold incorporated in the Berlin criteria and adopted in the NHANES study, but also introduced an upper limit of 4 h, to exclude all-day stiffness, which is more likely to be a feature of nociplastic pain than inflammation [31].

The results from this study may have implications for treatment of MC, which to date remains a controversial field, with at best modest treatment effects. Investigated treatments include epidural steroid injections, intra-discal steroid injections, spinal fusion, disc replacement, exercise therapy [32, 33], intraosseous basivertebral nerve ablation [34–36], intravenous tumor-necrosis factor inhibitors [37] and probiotics [38], as well as antibiotics [39, 40], a treatment option which remains controversial. While some interventional studies have investigated the effect of a range therapeutic approaches on biomarkers of inflammation [41, 42], to date no interventional study has incorporated an assessment of inflammatory symptoms. Combining inflammatory symptoms with MC into an inflammatory phenotype may provide greater predictive power than relying on MC alone. A fundamental limitation of current treatments is that they cannot be specifically targeted to the underlying causes of inflammation, which, while theories abound, are unknown.

This study has several limitations. First, the imaging features included in the comprehensive score applied in the comeBACK study was not specifically designed to capture inflammatory lesions. A score specifically tailored to inflammatory imaging lesions may have included enthesitis of the insertion sites of the interspinous ligaments and annulus fibrosus, that are plausibly associated with inflammation and may affect the relationship between inflammatory symptoms and Modic lesions. Second, the Modic classification criteria may not accurately reflect the extent of bone marrow inflammation, which is the nociceptive stimulus responsible for pain. While on average MC Type 2 lesions likely have less inflammation than Type 1, there may be sufficient inflammation with some Type 2 lesions to cause inflammatory pain. Third, the ARQ, the survey our IBP items were drawn from, was developed specifically for determining the prevalence of IBP in the population, using criteria from several different IBP classification systems. These systems were developed to either guide referrals from primary care to Rheumatology for evaluation of suspected axSpA, or to assist Rheumatologists in diagnosing axSpA; as such, they may not be optimal for identifying a nsCLBP subgroup with spinal inflammation. Furthermore, it is possible that non-spinal co-morbidities associated with systematic inflammation may influence IBP symptoms, and it cannot be excluded that such conditions (e.g. fibromyalgia) may have been included as unmeasured confounders. Finally, our exploratory study with limited sample size was not designed to robustly answer more elaborate question about the complex relationships among (these highly inter-correlated) imaging findings, IBP features and other dimensions of LBP, such as pain intensity or frailty and as such, our results require further confirmatory investigations.

In conclusion, the findings from this exploratory study mark a further step in identifying an inflammatory phenotype within nsCLBP, connecting inflammatory symptoms with inflammatory imaging findings. We found plausible positive associations between endplate erosion and Modic type 1 changes, and both lesions showed especially strong associations with morning stiffness and worsening with rest. Patients with an inflammatory phenotype may benefit preferentially from interventions, such as medications and injections, directed specifically towards inflammation. Pending confirmative results of our exploratory findings, future work on the definition of an inflammatory phenotype of nsCLBP could incorporate serum markers of inflammation and a more refined approach to identifying inflammatory lesions on MRI. Ultimately, the utility of defining a degenerative inflammatory phenotype, characterized by MC and/or endplate erosions and inflammatory symptoms, will need to be determined by clinical trials.

## Supplementary Information

Below is the link to the electronic supplementary material.

Supplementary Material 1

Supplementary Material 2

Supplementary Material 3

## Data Availability

The clinical research data supporting this research is publicly available (https://search.vivli.org/studyDetails/fromSearch/7a7b72f2-7086-4344-89ad-957c641c8c6c), or will be made available if still under embargo at the time of data request.
