# Supplementary material for: Association between MRI findings and inflammatory symptoms in non-specific chronic low back pain
Source: Eur Spine J. 2025 Oct 22;34(12):5530–8. doi: 10.1007/s00586-025-09492-7 (PMC12715048; doi:10.1007/s00586-025-09492-7)
Supplement: Supplementary file 1 — Supplementary Material 1 [file 586_2025_9492_MOESM1_ESM.pdf]

Inflammatory low back pain (IBP) measurements

Inflammatory symptoms were measured using items from a questionnaire administered in the 2009-10 National Health and Nutrition Examination Survey (NHANES), designed to provide population prevalence estimates for IBP (Appendix A) [1]
Responses from the questionnaire were recoded to define four binary (Yes/No) inflammatory symptom variables as detailed in *italics*.

Survey questions:

- When you wake up from up from sleep how long do you have stiffness? Would you say...
  - less than 10 minutes
  - 10 to 30 minutes
  - 31 to 60 minutes
  - more than 1 but less than 4 hours
  - more than 4 hours?
  - DON'T HAVE MORNING STIFFNESS
  - DON'T KNOW

  Yes= 31 to 60 minutes, more than 1 but less than 4 hours, otherwise No

- Does your back pain **usually** get better when you either walk or stretch for a half hour?
  - Yes
  - No
  - Don't do these activities
  - Don't know

  Yes= Yes, otherwise No.

- What usually happens to your low back pain over the time that you sleep or lay down?
  - Increases
  - Decreases
  - Stays the same
  - It varies, no pattern
  - Don't have sleep or rest pain
  - Don't know

  Yes= increases, otherwise No.

- Do you often wake up from sleep because of back pain?
  - Yes
  - No
  - Don't know

  Yes= Yes, otherwise No.

1. Weisman MH, Witter JP, Reveille JD. The prevalence of inflammatory back pain: population-based estimates from the US National Health and Nutrition Examination Survey, 2009-10. Ann Rheum Dis. 2013;72:369–73.
