# Supplementary material for: Association between MRI findings and inflammatory symptoms in non-specific chronic low back pain
Source: Eur Spine J. 2025 Oct 22;34(12):5530–8. doi: 10.1007/s00586-025-09492-7 (PMC12715048; doi:10.1007/s00586-025-09492-7)
Supplement: Supplementary file 2 — Supplementary Material 2 [file 586_2025_9492_MOESM2_ESM.docx]

**Appendix 3. Association of IBP and clinical features**. Results of logistic regression analyses, with IBP feature as outcome, and clinical features as predictors (all in the same model). Statistically significant results (p<0.05) are printed in bold and marked with as asterisk (*).

|  | | **Female sex** | **Age** | **BMI** | **Pain intensity** |
| --- | --- | --- | --- | --- | --- |
| **IBP feature [OR, 95%CI, p]** | Morning stiffness | 0.73  (0.45; 1.17)  P=0.703 | 0.99  (0.98; 1.01)  p=0.188 | 1.00  (0.96; 1.05)  p=0.190 | 1.04  (0.92; 1.19)  p=0.508 |
|  | Improvement with exercise | 0.76  (0.41; 1.40)  P=0.382 | **0.98***  **(0.96; 1.0)**  **p=0.030** | 0.97  (0.92; 1.03)  p=0.364 | 0.95  (0.81; 1.12)  p=0.540 |
|  | Worsening with rest | 0.49  (0.07; 3.41)  P=0.468 | 0.88  (0.51; 1.53)  p=0.655 | 0.96  (0.91; 1.02)  p=0.219 | 1.04  (0.90; 1.20)  p=0.625 |
|  | Waking from pain | 1.62  (1.00; 2.65)  P=0.052 | 1.00  (0.99; 1.02)  p=0.244 | 1.02  (0.97; 1.07)  p=0.517 | **1.28***  **(1.13; 1.47)**  **p<0.001** |
|  | Younger than 40 years old at onset | 1.10  (0.61; 1.99)  P=0.762 | **0.90***  **(0.87; 0.92)**  **p<0.001** | 0.99  (0.93; 1.05)  p=0.708 | 1.07  (0.92; 1.25)  p=0.412 |
|  | Insidious onset | 1.39  (0.84; 2.32)  P=0.884 | **1.02***  **(1.00; 1.04)**  **p=0.017** | 0.95  (0.90; 1.00)  p=0.062 | 1.10  (0.96; 1.26)  p=0.197 |
