# Supplementary material for: Association between MRI findings and inflammatory symptoms in non-specific chronic low back pain
Source: Eur Spine J. 2025 Oct 22;34(12):5530–8. doi: 10.1007/s00586-025-09492-7 (PMC12715048; doi:10.1007/s00586-025-09492-7)
Supplement: Supplementary file 3 — Supplementary Material 3 [file 586_2025_9492_MOESM3_ESM.pdf]

Table 1: MRI acquisition protocol

Sequence	Standard SAG T2-fs	Standard SAG T2	Standard SAG T1	Standard AX T2	Standard AX T1	Pelvis SAG T1	SI Joint COR T1
Patient Position	Head first, supine	Head first, supine	Head first, supine	Head first, supine	Head first, supine	Head first, supine	Head first, supine
Plane	SAG	SAG	SAG	AX	AX	SAG	COR
Refocus Flip Angle	120	120	110	120	110	115	115
TE [ms]	60	60	14.1	60	14.1	14.1	14.1
TR [ms]	4551	5,035	869	8,414	594	720	595
ETL	16	18	6	16	6	6	6
rBW [kHz/0.5FOV]	50	50	50	50	50	50	50
FOV (cm)	26	26	26	18	18	30	30
Slice Thickness [mm]	3.0	3.0	3.0	4.0	4.0	8.0	4.0
Slice Spacing [mm]	3.0	3.0	3.0	5.0	5.0	10	5.0
Freq. Matrix	384	384	384	288	288	320	384
Phase Matrix	224	224	224	192	192	160	224
Freq DIR	A/P	A/P	A/P	R/L	R/L	A/P	R/L
NEX averages	3.0	3.0	2.0	2.0	2.0	2.0	2.0
Fatsat	On	Off	Off	Off	Off	Off	Off
Acceleration	Off	Off	Off	Off	Off	Off	Off
# Slices	24	24	24	42	42	36	20
time	3:16	3:16	3:34	3:31	3:47	2:06	1:42

Table 2: MRI scoring protocol

	0	1	2	3	4	5	6
**BMIC (Modic Changes)**							Pulse Sequence
BMIC	Absent	Present					
Type		hypointense T1,<br>hyperintense T2<br>hyperintense T2-fs	hyperintense T1,<br>hyperintense T2<br>Iso- hypointense T2-fs	hypointense T1,<br>hypointense T2<br>hypointense T2-fs			SAG T2<br>SAG T1<br>SAG T2-fs
Height		localized to endplate only	less than 25% of vertebral body height	25 to 50% of vertebral body height	more than 50% vertebral body height		SAG T2<br>SAG T1<br>SAG T2-fs
Area (largest diameter in a single section)		less than 25% of endplate area	25 to 50% of endplate area	more than 50% of endplate area			SAG T2<br>SAG T1<br>SAG T2-fs

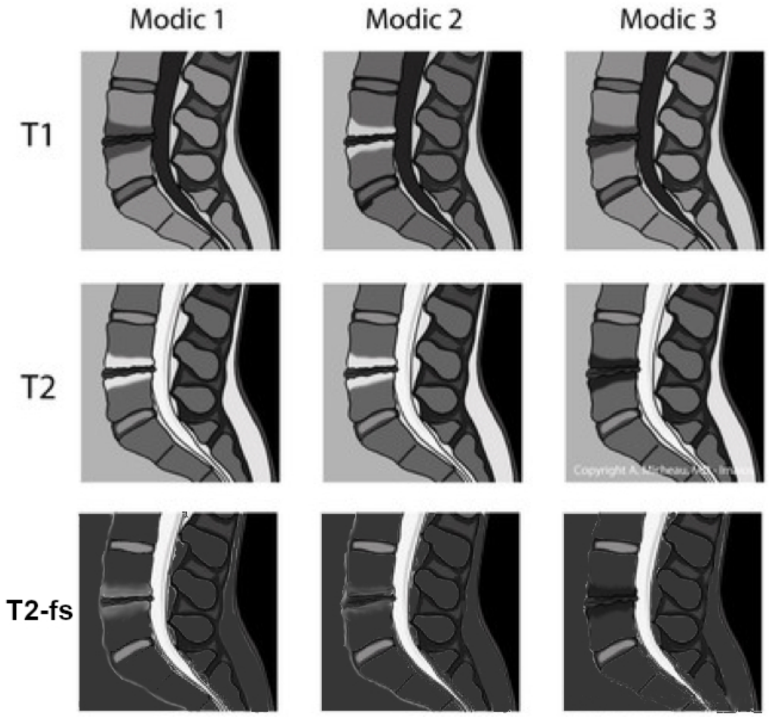

	0	1	2	3	4	5	6
**Endplate Defect**							Pulse Sequence
Endplate Defect	Absent	Present					
Type		Degenerative (erosive osteochondrosis)	Scheuermann Variant (Schmorl's nodes) Wedge shape deformity and increased sagittal diameter	Osteoporotic (normal sagittal diameter)	Other		SAG T1<br>SAG T2
Shape		Irregular, diffuse	Schmorl's node subchondral cyst	sharp, angular, focal	Other		SAG T1<br>SAG T2
Size (largest diameter in a single section)		less than 1/3 endplate area	between 1/3 and 2/3 endplate area	more than 2/3 endplate area			SAG T1<br>SAG T2
Depth (largest diameter in a single section)		less than 25% of vertebral body height	25 to 50% of vertebral body height	more than 50% vertebral body height			SAG T1<br>SAG T2

Erosive Osteochondrosis

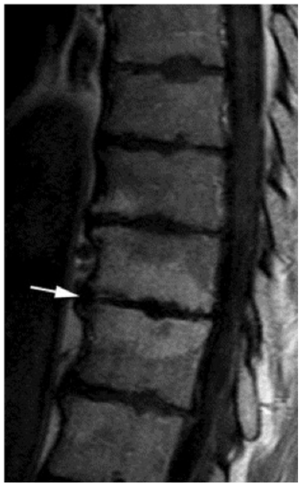

Scheuermann Disease

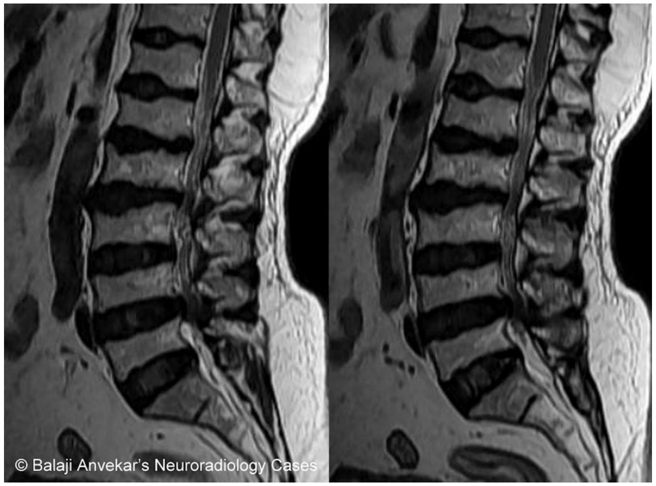

Osteoporotic Fractures

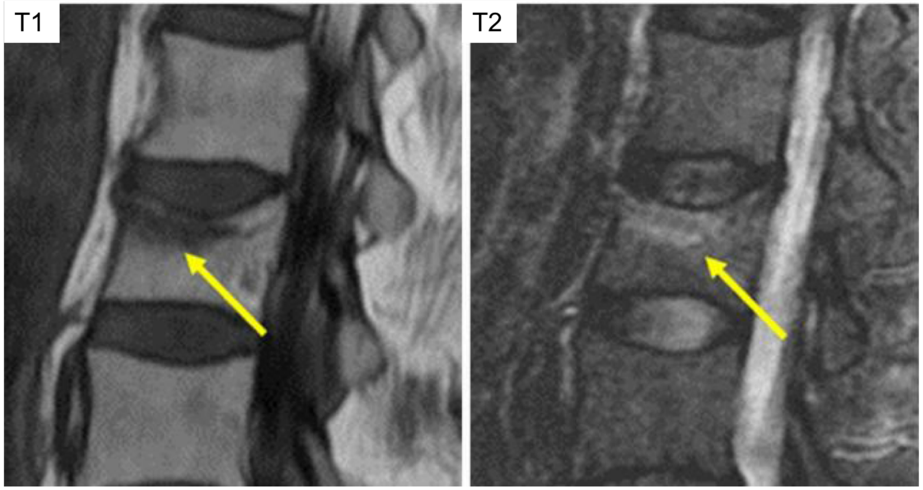

	0	1	2	3	4	5	6
**Disc**							Pulse Sequence
DDD (Pfirrmann)		homogeneous, hyperintense, normal height	inhomogeneous, hyperintense, normal height clear distinction annulus vs. nucleus	inhomogeneous, grey, normal height no clear distinction between annulus and nucleus	Inhomogeneous, grey to black, normal height to moderate loss no distinction between annulus and nucleus	Inhomogenous, black, > 50% height loss	SAG T2
DH (disc height)	less than 10% loss (mild)	10%-50% loss (moderate)	>50% loss (severe)				SAG T2
Disc Herniation	normal	bulge	protrusion	extrusion			SAG T2-fs<br>AX T2
Protrusion/extrusion location	Central	Subarticular	Foraminal	Extraforaminal			SAG T2
Protrusion/extrusion location Left/Right	Left	Right					SAG T2
AF (Annular Fissure)	Absent	Present					SAG T2
AF location	Central - posterior	Left/subarticular	Right/subarticular				SAG T2
Nerve Root Involvement (NRI)	No nerve root contact	Nerve root – left contact without deviation	Nerve root -left deviation and compression	Nerve root -right–contact without deviation	Nerve root -right deviation and compression	Bilateral	SAG T1<br>SAG T2<br>AX T2

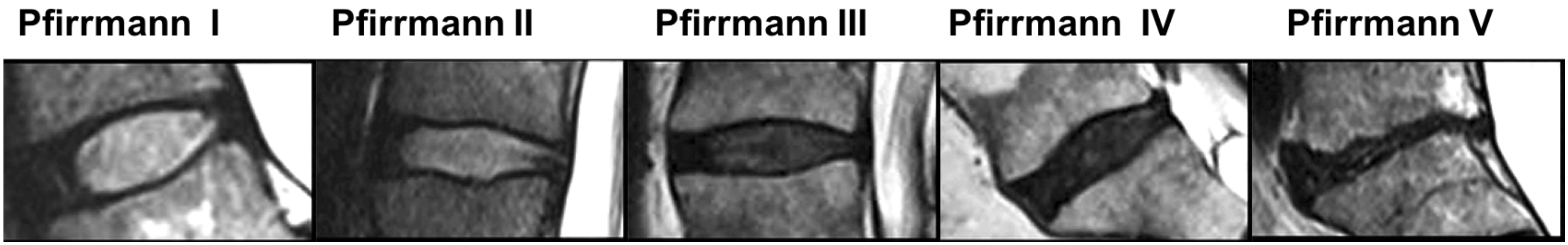

	0	1	2	3	4	5	6
**Facet**							Pulse Sequence
FJ OA – most severe joint	None	Narrowing, small osteophytes or hypertrophy	Narrowing, moderate osteophytes or hypertrophy	Narrowing, large osteophytes or severe hypertrophy			SAG T2-fs<br>AX T2<br>Ax T1
FJ Fluid	absent	present					Ax T2-fs

Grade 0 | Grade 1 | Grade 2 | Grade 3

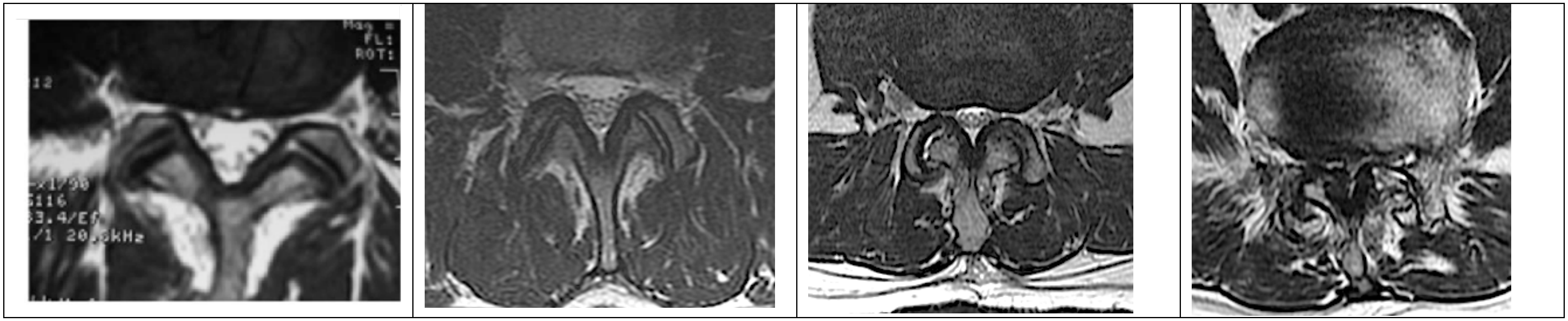

	0	1	2	3	4	5	6
**Stenosis**							Pulse Seq.
Central canal (CCS)	No thecal sac constriction	Mild constriction, minimal loss of CSF around rootlets	CSF diminished but present	Complete loss of CSF			SAG T2-fs<br>AX T2
Congenital narrowing spinal canal (smaller 13 mm- ap diameter- L2-5) at the level of the pedicles	No	Yes				Singh et al. Spine J. 2005 Nov-Dec; 5(6):615-22.	SAG T2-fs<br>AX T2
LRS (lateral recess stenosis) related to disc, facet arthropathy	No nerve root contact	Nerve root contact without deviation	Nerve root compression				SAG T2-s<br>AX T2
LRS (lateral recess stenosis) location	Left	Right	Bilateral				SAG T2-s<br>AX T2
FS (foraminal stenosis)	normal epidural fat	Mild - slight deformity of epidural fat, still completely surrounding nerve root	Moderate- marked deformity of epidural fat, only partially surrounding nerve root	Severe- obliteration of epidural fat			SAG T1<br>SAG T2-fs
FS (foraminal stenosis) L/R/Bilateral	Left	Right	Bilateral				SAG T1<br>SAG T2-fs
Lumbo-sacral segmentation abnormality	None	Large transverse processes (>2.1cm)	Large transverse process articulating with the sacrum	Large transverse process fused with sacrum		Nardo L et al. Radiology. 2012; 265: 497-503	SAG T1<br>SAG T2-fs<br>COR T1 pelvis
Lumbo-sacral segmentation abnormality - laterality	Bilateral	Left	Right	Asymmetric		Nardo L et al. Radiology. 2012;265: 497	SAG T1<br>SAG T2-fs<br>COR T1 pelvis
Sacro-iliac joints	Normal	Abnormal					SAG T2-fs<br>COR T1
Sacro-iliac joints Joints	Normal	Degenerative	Erosions				COR T1
Sacro-iliac joints Bone marrow	Normal	Bone marrow edema	Fatty transformation				SAG T1<br>COR T1
Sacro-iliac joints Bone	Normal	Insufficiency fractures	Osteitis condensans ilii	Other			SAG T1<br>COR T1
